# A big picture of a small brain

**DOI:** 10.7554/eLife.05580

**Published:** 2014-12-24

**Authors:** Leslie C Griffith

**Affiliations:** Department of Biology, the Volen National Center for Complex Systems and the National Center for Behavioral Genomics, Brandeis University, Waltham, United Statesgriffith@brandeis.edu

**Keywords:** mushroom body, olfactory learning, associative memory, behavioral valence, sleep, *D. melanogaster*

## Abstract

A detailed map of the neurons that carry information away from the mushroom bodies in the brains of fruit flies has improved our understanding of the ways in which experiences can modify behaviour.

**Related research articles** Y Aso, D Hattori, Y Yu, RM Johnston, NA Iyer, TTB Ngo, H Dionne, LF Abbott, R Axel, H Tanimoto, GM Rubin. 2014. The neuronal architecture of the mushroom body provides a logic for associative learning. *eLife* **3**:e04577. doi: 10.7554/eLife.04577

Y Aso, D Sitaraman, T Ichinose, KR Kaun, K Vogt, G Belliart-Guérin, PY Plaçais, AA Robie, N Yamagata, C Schnaitmann, WJ Rowell, RM Johnston, TTB Ngo, N Chen, W Korff, MN Nitabach, U Heberlein, T Preat, KM Branson, H Tanimoto, GM Rubin. 2014. Mushroom body output neurons encode valence and guide memory-based action selection in *Drosophila*. *eLife* **3**:e04580. doi: 10.7554/eLife.04580

**Image** High-resolution image of the different types of mushroom body output neurons in the brain of a fruit fly

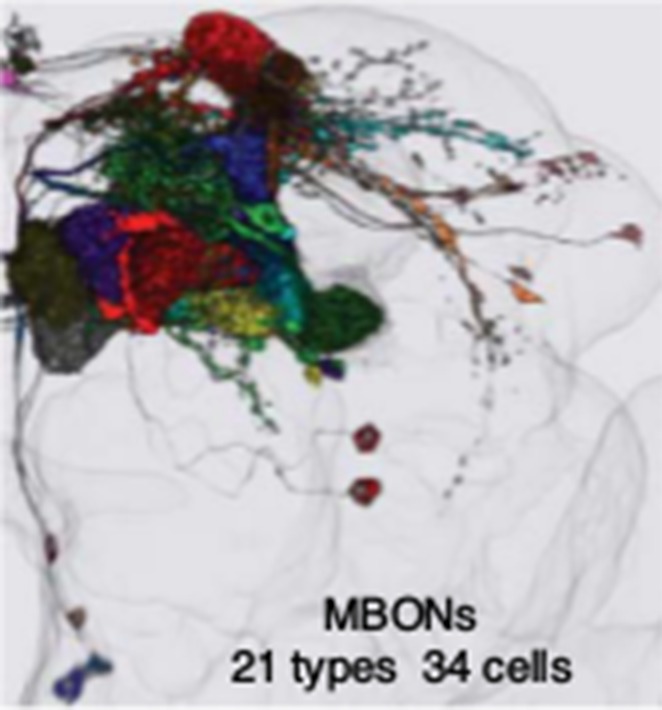

The fruit fly *Drosophila melanogaster* has been widely studied in the quest to understand how the function of the brain is modified in response to experience. Its complex behaviors and the ease with which it can be genetically manipulated allowed researchers to obtain the first insights into the genes that are involved in learning and memory in animals (reviewed in Kahsai and Zars, 2011). Many of the roles that the products of these genes play in learning in *Drosophila* were found to occur in a structure called the mushroom body. Over the next few decades, researchers developed a detailed understanding of how the mushroom body acquires and processes information to form memory (reviewed in Dubnau and Chiang, 2013).

What was completely missing, however, was any idea of how information gets out of the mushroom body to influence behavior. Low-resolution microscopy images of the structure as a whole were uninformative. The major class of mushroom body neurons—the Kenyon cells—had no obvious projections to carry information out of that brain area. A few mushroom body output neurons (MBONs) had been identified (e.g. Séjourné et al., 2011; Pai et al., 2013; Plaçais et al., 2013), but the total number of MBONs and their functional organization was unknown. Now, in two massive papers published in *eLife,* a group lead by Yoshinori Aso and Gerald Rubin of the Janelia Research Campus has filled this void (Aso et al., 2014a, 2014b). These two papers provide a complete picture of the cells that make up the mushroom bodies, including a full catalog of the MBONs, and they also suggest a model for how the mushroom body can integrate experiences and influence behavior.

The first paper—which includes co-authors at Janelia, Columbia University, Tohoku University and the Max Planck Institute for Neurobiology (MPIN)—presents the most detailed reconstruction of a single brain area ever done using light-based microscopy (Aso et al., 2014a). To make sure that they did not miss any mushroom body components, Aso et al. started by performing a visual screen of a collection of fruit fly lines that produce a fluorescent protein in different large, but anatomically distinct, subsets of neurons. Many of these lines contained small subsets of mushroom body cells on a background of non-mushroom body cells. To get more specific markers, they developed an novel genetic strategy to create new lines – called split-GAL4 lines - that marked only the few mushroom body cells in the initial lines. To ensure this new collection was complete, Aso et al. used an independent live-labeling method to mark the cells that had mushroom body projections. They compared these results to their split-GAL4 collection, which validated the marker lines and allowed them to fill in gaps.

The mushroom body contains three lobes (Figure 1) and Aso et al. found that these lobes contain processes from about 2200 neurons. There are seven types of Kenyon cells (∼2000 neurons), 21 types of MBONs (34 neurons total; Figure 2) and 20 types of cells that use dopamine as a neurotransmitter (called dopaminergic neurons; ∼130 cells) that feed into the structure. Moreover, each of these three classes of neuron was divided into multiple types based on the presence of that group of neurons in multiple marker lines, their morphology, and where they project to in the brain. These similarities suggest that each neuron type likely has a common developmental origin and function.

**Figure 1.**
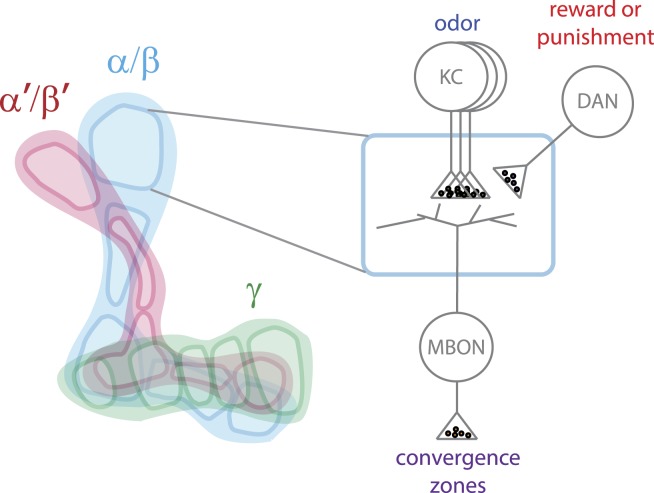
Model of how information is processed in the mushroom body to enable it to alter behavior. The mushroom body has three lobes (left) called α/β (blue), α′/β′ (pink) and γ (green). Each lobe is made up of projections from many Kenyon cells (KC) and is divided into five compartments (shown by the dark outlines). Each compartment (right) is defined by the projections from a limited number of dopaminergic neurons (DANs) and mushroom body output neurons (MBONs). The Kenyon cells carry sparse information about odor, while the dopaminergic neurons signal a reward or a punishment. The MBONs carry information away from the mushroom body to convergence zones in order to alter behavior. The Kenyon cells of each lobe extend through all the compartments of that lobe and form synapses with all the MBONs. However, each compartment contains a unique set of dopaminergic neurons and MBONs: this allows the information about odor to be used by several MBONs and to be modified by multiple types of dopaminergic neurons.

**Figure 2.**
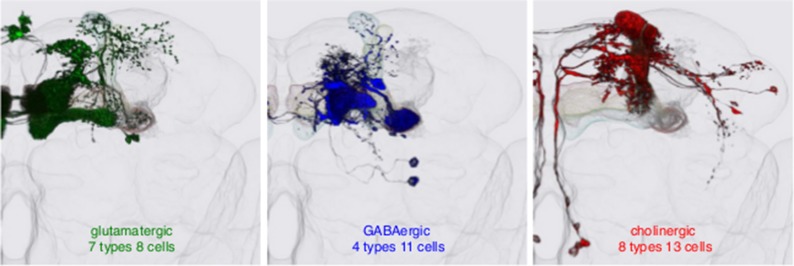
The many types of mushroom body output neurons (MBONs). Maps of the fly brain from Aso et al. (2014a) showing the locations of different types of MBONs, split into three groups according to the neurotransmitter chemical that they release. There are 21 types of MBONs in the fly brain: seven of these types release glutamate (shown in green in the left panel); four types release gamma-aminobutyric acid (GABA; shown in blue in the middle panel); and eight types release acetylcholine (shown in red in the right panel). The neurotransmitters released by the remaining two types are not known.

A complete parts list is a virtue when you want to know how a machine works, but it is more important to understand how those parts fit together. Using the split-GAL4 lines, Aso et al. painstakingly mapped the location of each cell type onto a reference brain template. The different types of MBONs have dendrites that project to limited areas of the mushroom body neuropil—an area that contains a large number of connections between neurons—and they divide the mushroom body into 15 compartments (Figure 1). Each type of dopaminergic cell connects to one or two of these compartments.

When the fly detects an odor—or another stimulus from the environment—sensory neurons activate Kenyon cells, which carry the information to the MBONs. The dopaminergic neurons—which signal a reward or punishment—connect to the synapses between the Kenyon cells and MBONs to modify their output. This creates a series of units within the mushroom body that can link sensory information with experience. There are also some recurrent connections, which may allow more complex processing of the information.

The ∼2000 Kenyon cells—which carry information that is both sparse and unstructured—form synapses with just 34 MBONs. This means that the information carried out of the mushroom body is limited and is unlikely to be a detailed representation of the sensory experience. Ultimately, the MBONs connect to five regions—called convergence zones—outside the mushroom bodies, where the information they carry is re-combined with information from sensory neurons. These data put important constraints on models of how the mushroom body works. They suggest that it acts as an indicator of the nature or ‘valence’ (positive or negative) of the association that has been learned, which is then used alongside other information to guide behavior.

In the second paper, Aso, Rubin and co-workers from Janelia, Yale School of Medicine, Tohoku, MPIN and the ESPCI in Paris tested this model by characterizing the roles of the different types of MBONs (Aso et al., 2014b). The split-GAL4 lines generated to mark these cells were used to drive the expression of genes that could turn the activity of neurons on or off when exposed to light or heat. Aso et al. started with the most basic question: is the activation of specific MBONs innately attractive or aversive? If MBONs carry information about valence, activating them should influence the fly's immediate behavioral decisions. One way to answer this question is to watch where the flies move in an arena in which the experimenters can control the conditions in the different regions of the arena. Aso et al. divided their arena into quadrants that were either dark, or lit with a wavelength of light that would activate certain MBONs. It was found that activating individual MBONs in the flies could alter the direction of movement and cause them to develop preferences for particular quadrants. The effects were additive: in other words, activating two MBONs that were both innately attractive had a bigger impact on the behavior of the fly than just activating one.

Aso et al. then worked their way through a large number of other behaviors to explore different sensory systems and different time scales of memory. They found that each behavior could be blocked by inhibiting the release of neurotransmitters in a particular type of MBONs. The fact that some behaviors involved attractive experiences, while others involved aversive experiences, made it possible to put the MBONs into groups according to valence. Moreover, there appears to be a relationship between the valence and the type of neurotransmitter involved: MBONs that use glutamate as a neurotransmitter are required for wakefulness and for remembering aversive cues, while MBONs that use acetylcholine and gamma-aminobutyric acid (GABA) promote sleep and are required for remembering attractive cues**.**

Taken together, the extensive data on the anatomy of the mushroom body and the behavioral impact of altering MBON activity are consistent with MBONs encoding valence. The additive nature of their effects, and the fact that individual cells of opposing valence project to the same regions of the brain, suggest that it is the balance of outputs from the MBONs that determines the effect of the mushroom body on behavior. Since the sensory information entering it is sparse, the mushroom body has the potential to process many experiences at the same time, and then feed them into the MBON network to derive a customized output that reflects the current situation of that animal. This argues that the general role of the mushroom bodies is to bias decision-making rather that drive specific behaviors.

These two papers are very important for our understanding of behavior, but they are also quite unusual. The scope of the studies is huge. The decision to present the data as complete stories, with 22 figures in the first paper and 15 figures in the second, rather than divide them into 1500 word sound bites, was a good one for the field. The first paper in particular is encyclopedic in its review of previous work and is likely to become a reference document for those interested in the mushroom bodies. The maps and tools that the studies generated will enable deeper investigations into how animals make choices. The small brain of the fly is having a big impact on how we understand decision-making.

## References

[bib1] Aso Y, Hattori D, Yu Y, Johnston RM, Iyer N, Ngo TB, Dionne H, Abbott LF, Axel R, Tanimoto H, Rubin G (2014a). The neuronal architecture of the mushroom body provides a logic for associative learning. eLife.

[bib2] Aso Y, Sitaraman D, Ichinose T, Kaun KR, Vogt K, Belliart-Guérin G, Plaçais PY, Robie AA, Yamagata N, Schnaitmann C, Rowell WJ, Johnston RM, Ngo TTB, Chen N, Korff W, Nitabach M, Heberlein U, Preat T, Branson KM, Tanimoto H, Rubin GM (2014b). Mushroom body output neurons encode valence and guide memory-based action selection in Drosophila. eLife.

[bib3] Dubnau J, Chiang AS (2013). Systems memory consolidation in Drosophila. Current Opinion in Neurobiology.

[bib4] Kahsai L, Zars T (2011). Learning and memory in Drosophila: behavior, genetics, and neural systems. International Review of Neurobiology.

[bib5] Pai TP, Chen CC, Lin HH, Chin AL, Lai JS, Lee PT, Tully T, Chiang AS (2013). Drosophila ORB protein in two mushroom body output neurons is necessary for long-term memory formation. Proceedings of the National Academy of Sciences of USA.

[bib6] Plaçais PY, Trannoy S, Friedrich AB, Tanimoto H, Preat T (2013). Two pairs of mushroom body efferent neurons are required for appetitive long-term memory retrieval in Drosophila. Cell Reports.

[bib7] Séjourné J, Placais PY, Aso Y, Siwanowicz I, Trannoy S, Thoma V, Tedjakumala SR, Rubin GM, Tchenio P, Ito K, Isabel G, Tanimoto H, Preat T (2011). Mushroom body efferent neurons responsible for aversive olfactory memory retrieval in Drosophila. Nature Neuroscience.

